# Pediatric Tracheotomy: Modern Surgical Techniques, Challenges, and Clinical Considerations

**DOI:** 10.3390/children12050637

**Published:** 2025-05-15

**Authors:** Stoyan S. Markov, Petya P. Markova, Kalina I. Madzarova-Nikolova

**Affiliations:** 1 Department of Otorhinolaryngology, Medical University of Plovdiv, 4002 Plovdiv, Bulgaria; kalina.madzarova@mu-plovdiv.bg; 2 Department of Otorhinolaryngology, “St. George” University Hospital Plovdiv, 4001 Plovdiv, Bulgaria; 3 Department of Pediatrics, Medical University of Plovdiv, 4002 Plovdiv, Bulgaria; petya.markova@mu-plovdiv.bg; 4 Department of Pediatrics, “St. George” University Hospital Plovdiv, 4001 Plovdiv, Bulgaria

**Keywords:** pediatric tracheotomy, tracheotomy surgical approach, types of tracheotomy

## Abstract

**Introduction**: Tracheotomy as a surgical procedure has existed and evolved since ancient times. In modern medicine, surgical techniques for performing this procedure in adults and children have reached a high level of perfection. However, pediatric tracheotomy remains a challenging surgical intervention, performed in only a limited number of centers by a small number of surgeons. This is due to several unresolved issues related to the procedure—such as indications, timing, decannulation protocols, and the care of tracheotomized children—which are still usually left to the individual judgment of the physician. Additionally, there is a significant psychological barrier associated with performing tracheostomy in a child (particularly in those under the age of one). **Aim**: This article aims to present in detail the modern surgical approach to performing tracheotomy in pediatric patients, examine the different types of tracheotomy, and highlight its specific features related to the anatomical differences between children and adults. **Discussion**: After the decision to perform a tracheotomy is made, the exact surgical technique and type of tracheostomy to be created are selected alongside the patient’s preoperative preparation. Factors such as the child’s age, the surgeon’s experience, and the underlying disease for tracheotomy play crucial roles in determining the appropriate approach. **Conclusions**: Pediatric tracheotomy has undergone significant development over the years. Nowadays, the exact type of surgical intervention depends on the individual needs of young patients.

## 1. Introduction

Originating in ancient times as an emergency surgical intervention to resolve acute asphyxiation, pediatric tracheotomy has undergone significant evolution over the years. In modern medicine, it is primarily performed as a planned procedure for children who represent a complex patient group requiring long-term mechanical ventilation and/or tracheobronchial toilet [1,2].

Despite the opinion of some historians that tracheotomy was performed as early as ancient Egypt, historical data suggest that the first tracheotomy was performed by Asclepius in ancient Rome in the 2nd century BCE. Centuries later, in the 16th century (1546), the Italian physician Antonio Musa Barsolva performed the first successful pediatric tracheotomy to overcome upper airway obstruction caused by tonsillar hypertrophy. Over the years, it has been used and refined by numerous surgeons—Nicolas Habicot, Bretonneau, Trousseau, Chevalier Jackson—and established as a valuable procedure when Galloway reported its successful use in aiding breathing for children with poliomyelitis during the poliomyelitis pandemic of the 1950s [1].

The main indications for performing a tracheotomy, both in children and adults, include overcoming airway obstruction, facilitating care for patients on long-term mechanical ventilation, protecting against aspiration in patients with impaired swallowing reflexes, preventing laryngotracheal stenosis in patients requiring prolonged intubation, and others [3].

This article aims to present in detail the modern surgical approach to performing tracheotomy in pediatric patients, examine the different types of tracheotomy, and highlight its specific features related to the anatomical differences between children and adults.

## 2. Specific Considerations for Performing Tracheotomy in Pediatric Patients

When planning to perform a tracheotomy in infants and children, it is important to consider the anatomical differences in the larynx between them and adults [4]. Both the anatomical and physiological characteristics of a child’s trachea, especially in babies, require specialized surgical techniques and adequate postoperative care (children are not simply small adults).

The main intraoperative difficulties are due to the following anatomical features:

1. The size of the hyoid bone in children often exceeds that of the thyroid cartilage, making the palpation of anatomical landmarks more challenging.
2. The thyrohyoid membrane is significantly shorter in infants.
3. The cricoid cartilage is the narrowest part of the airway in children, whereas the glottic opening is the narrowest in adults.
4. The pediatric trachea is shorter, narrower, and more compliant, increases the risk of tracheomalacia and airway collapse.
5. In infants and young children, the larynx is located higher in the neck (around C3–C4 compared to C5–C6 in adults), making access to the trachea more challenging.
6. The pediatric thyroid gland is proportionally larger and highly vascularized, increasing the risk of bleeding during surgery.
7. The supraglottis and subglottis mucosa are more relaxed in infants, making them more susceptible to swelling in the case of inflammation or injury.

## 3. Surgical Techniques for Performing Tracheotomy in Childhood

There are two main modern approaches to performing tracheotomy in both children and adults: classical and transcutaneous (percutaneous).

Percutaneous tracheostomy (PCT) was developed by Toye and Weinstein (1969) and has been applied in pediatric patients. Pasquale Ciaglia later described percutaneous dilational tracheostomy (PDT) and subsequently introduced the first kit for its performance. Over time, his technique underwent several modifications, incorporating bronchoscopy and ultrasound for enhanced precision and safety. This type of tracheotomy is considered safe and easy to perform, offering several advantages over classical tracheotomy; it does not damage the tracheal cartilage and provides better cosmetic outcomes. For these reasons, it has largely become the preferred technique for adult patients. Despite these benefits, this technique is rarely used in children due to concerns regarding the safety of the procedure and technical limitations, especially in infants and young children [5].

The difficulties in performing percutaneous tracheostomy at a young age are due to the following objective factors:

1. According to statistical data, between 40% and 50% of pediatric tracheostomies are performed in infants under one year age. They have extremely small airways, making the palpation of the anatomical landmarks very difficult. This, in turn, significantly complicates the accurate insertion of the needle, guidewire, dilators, and tracheostomy cannula through the soft tissues into the correct anatomical section of the neck.
2. The trachea in children is much more mobile and softer than the adult trachea. As a result, there is an increased risk of tracheal collapse when pressure is applied with the dilators, which in turn raises the risk of posterior tracheal wall damage.
3. In some cases, the underlying condition or disease requiring tracheostomy can also be a limiting factor. For example, in cases of subglottic stenosis, tracheal stenosis, or tracheomalacia, performing percutaneous tracheostomy in a narrowed tracheal lumen can be extremely challenging.
4. Accidental decannulation in the early postoperative period can be fatal due to the smaller tracheostomy opening and the absence of stay sutures, which are typically placed during a classical pediatric tracheotomy. These sutures help facilitate both the replacement of the tracheostomy cannula in the first few days after surgery and recannulation in the case of accidental decannulation.

Despite the difficulties described above, percutaneous tracheostomy in childhood is gradually gaining popularity. Nowadays, many authors consider the procedure safe for children over 10 years of age, and in some pediatric hospitals, it has been successfully performed on patients over 5 years of age. However, in all cases where this technique is used, experts strongly recommend performing it in an operating room under strict bronchoscopic guidance to prevent complications.

## 4. Classical Tracheotomy

Nowadays, the majority of pediatric tracheostomies are still performed using the classical open surgical technique, which has been refined over decades. Various modern surgical modifications have been introduced to minimize the risks and complications of the procedure. Most debates and innovations focus on aspects such as the type of tracheal incision, whether to create a tracheal flap, and whether to suture the trachea to the skin. Many authors recommend performing preoperative laryngotracheobronchoscopy to assess the condition of the airway and identify any additional pathology that may require treatment after tracheotomy [5].

**Figure 1:**
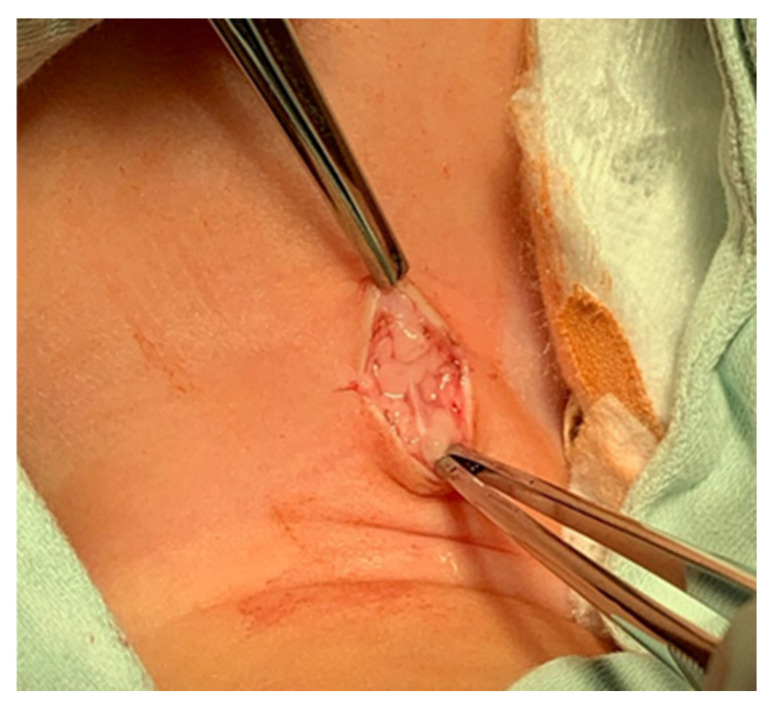
Neck midline incision.

**Figure 2:**
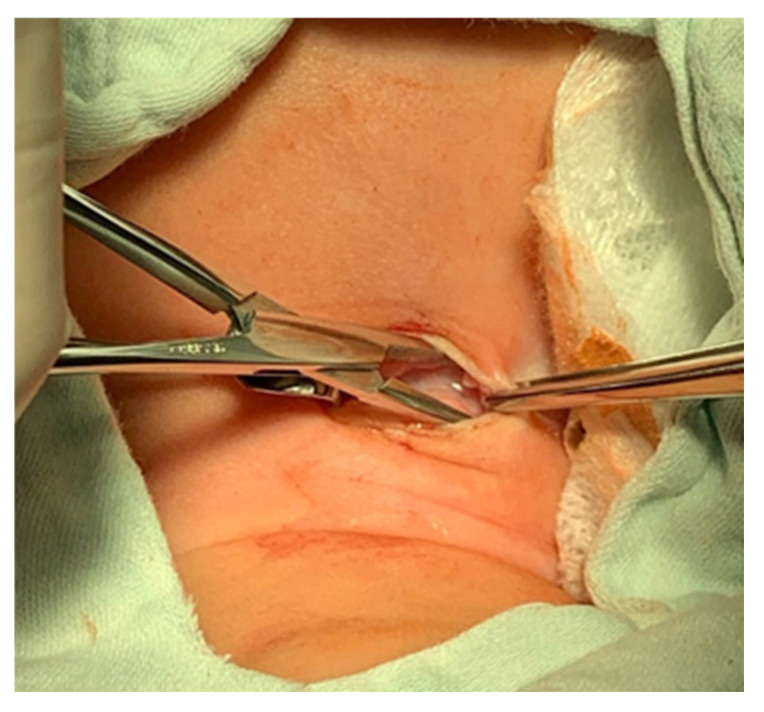
Subcutaneous tissue dissection.

**Figure 3:**
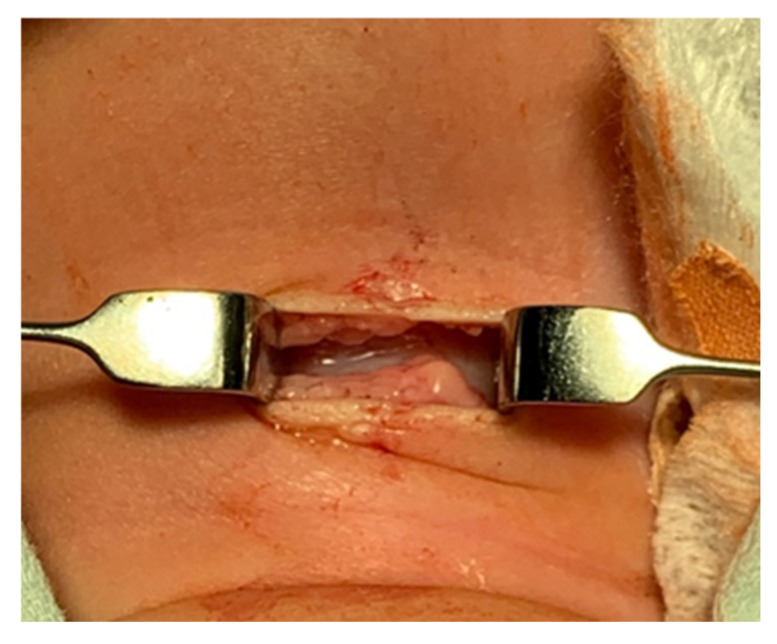
Platysma retraction.

**Figure 4:**
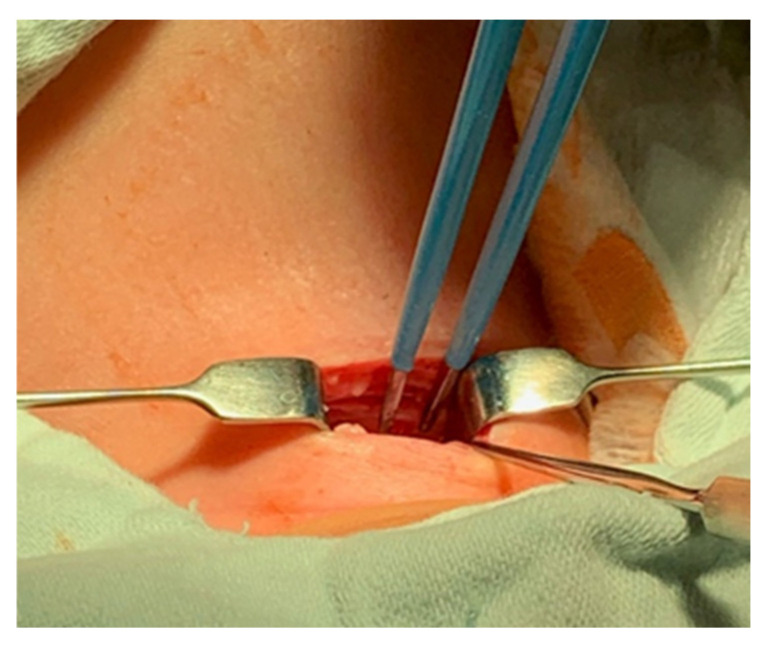
Bipolar cautery hemostasis.

**Figure 5:**
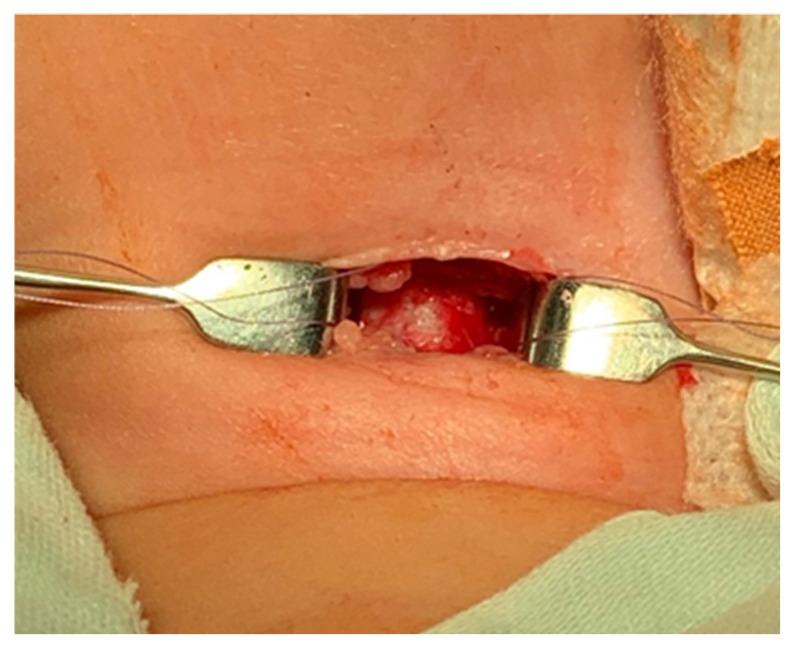
Stay sutures placement.

**Figure 6:**
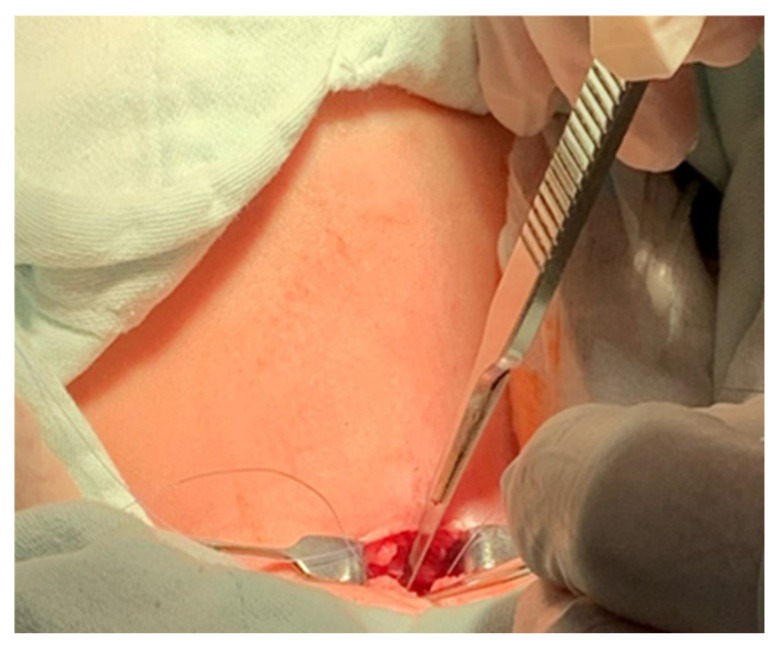
Tracheal incision.

**Figure 7:**
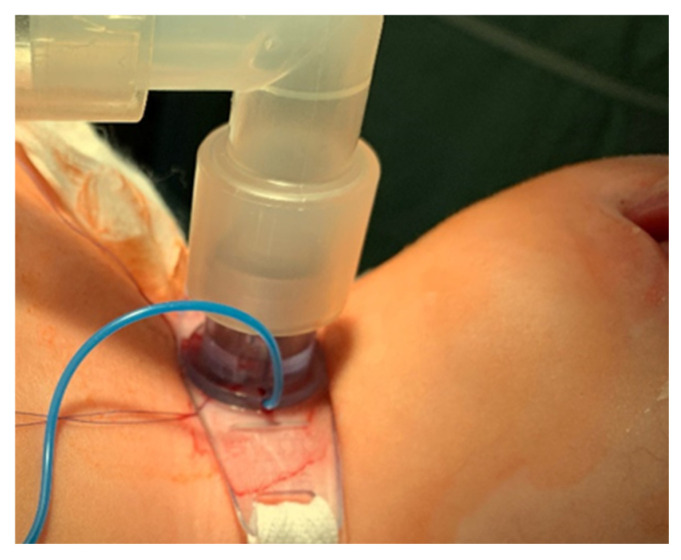
Cannula connection to the ventilator.

**Figure 8:**
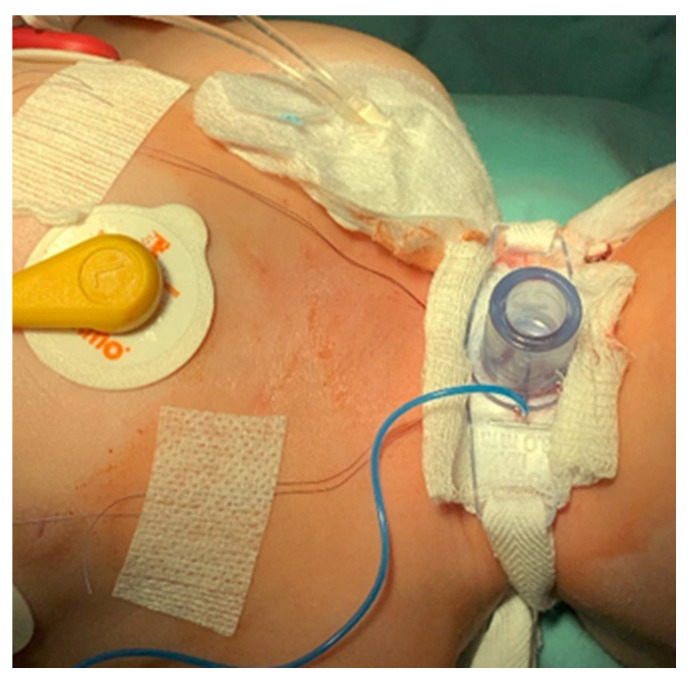
Stay suture attachment and labeling.

A pediatric tracheotomy can be stylized and represented as follows:

1. The child is placed in the supine position with the head in maximum deflection.
2. Careful palpation of the anatomical landmarks is performed to identify the cricoid cartilage and jugular notch [3].
3. Stepwise infiltration of the skin, subcutaneous tissue, and underlying soft structures was performed with a solution of local anesthetic and adrenaline to reduce intraoperative bleeding. If a tracheotomy is performed under local anesthesia (which is rare in pediatric patients), infiltration is usually multi-stage.
4. A midline incision is made on the neck, halfway between the cricoid cartilage and the jugular notch (Figure 1). The incision can be either horizontal or vertical, with a horizontal incision being used in most cases.
5. The subcutaneous tissue is carefully dissected (Figure 2).
6. The platysma is reached, and its muscles are separated and retracted using retractors (Figure 3).
7. Bipolar cautery is used during the procedure for hemostasis (Figure 4).
8. The pretracheal longitudinal muscles are exposed, separated along the midline of the neck, and retracted laterally.
9. If the thyroid isthmus covers the trachea and it is impossible to displace it cranially or caudally, it is clamped and cut. Since the thyroid gland is a well-vascularized parenchymal organ, cauterization is used for hemostasis, and the edges of the incision are almost always additionally sutured [3].
10. The anterior surface of the trachea is exposed along 3–4 rings.
11. The larynx and trachea are pulled upward and secured using a hook.
12. Some authors recommend the routine placement of two sutures vertically on either side of the future tracheal incision (stay sutures) to facilitate easier and faster recannulation in case of accidental decannulation, as well as during the cannula change in the first few days after surgery (Figure 5) [3].
13. A vertical or horizontal incision is made on the trachea, usually between the 2nd and 4th tracheal rings (Figure 6).
14. A tracheal dilator is used to expand the tracheal incision.
15. The endotracheal tube is identified, and after deflating the cuff, it is gradually pulled upward until positioned just below the vocal cords.
16. With the help of the guidewire, the cannula is inserted into the lumen of the trachea, after which the guidewire is removed.
17. The cannula is connected to the ventilator, and its cuff is inflated (Figure 7).
18. The endotracheal tube is not fully removed until the cannula is correctly positioned (its lower end should be 2–3 rings above the carina) [6] and the patient’s ventilation is adequate. This positioning is verified through fiberoptic endoscopic examination [3].
19. After confirming the correct position of the cannula, the hook and retractor are removed.
20. The cannula is secured around the patient’s neck with ties, and, in rare cases, it may also be sutured to the skin.
21. If stay sutures have been placed on the trachea, they are secured to the patient’s chest and are clearly labeled as “left” and “right.” (Figure 8). These sutures are typically removed during the first or second cannula changes.
22. A soft padding is placed under the cannula plate to prevent irritation or injury.

When performing tracheotomy in childhood, it is recommended to maintain spontaneous breathing in order to identify an acute airway problem. Excision of the peristomal adipose connective tissue and suturing of the edges of the tracheal opening to the skin for the purpose of “maturation of the tracheostomy” (maturation sutures), mainly when forming a permanent tracheostomy. has been recommended by numerous authors [7]. Preservation of the tracheal rings during the procedure is crucial for preventing suprastomal collapse and tracheomalacia [3].

The extensive sequence of steps described for performing pediatric tracheotomy can be followed primarily during planned procedures under general anesthesia. Naturally, in emergency situations, the sequence is shortened.

## 5. Permanent Tracheostoma Formation

There is a specific group of patients, both children and adults, indicated for tracheotomy, in whom the primary disease makes it clear, even preoperatively, that the created tracheostomy opening will have to stay for life. In childhood, these are primarily patients with severe neurological disorders [8]. Various techniques are used in these patients to create a permanent tracheostomy opening to facilitate care and prevent accidental decannulation. Due to the general aversion to resecting a part of the child’s anterior tracheal wall, most stomaplasty methods involve only suturing the skin around the edges of the tracheal opening.

**Figure 9:**
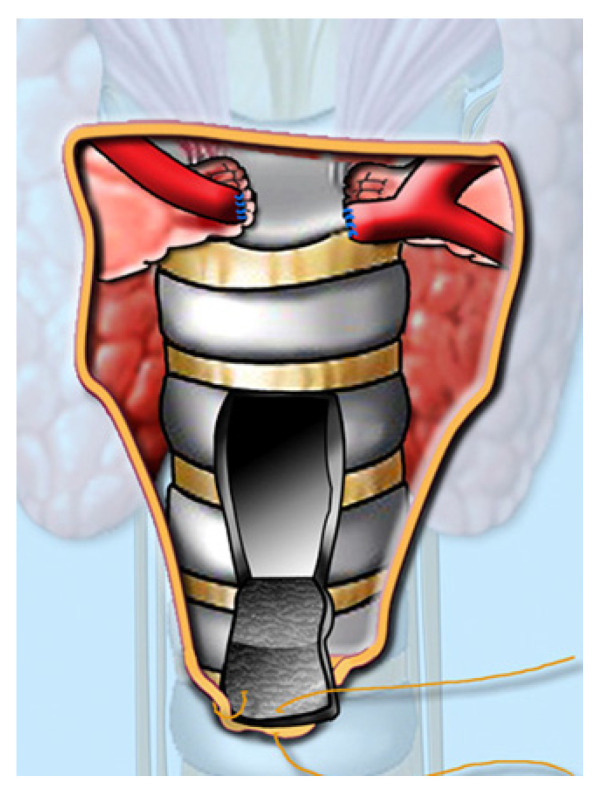
Bjork tracheostomy flap anterior view.

**Figure 10:**
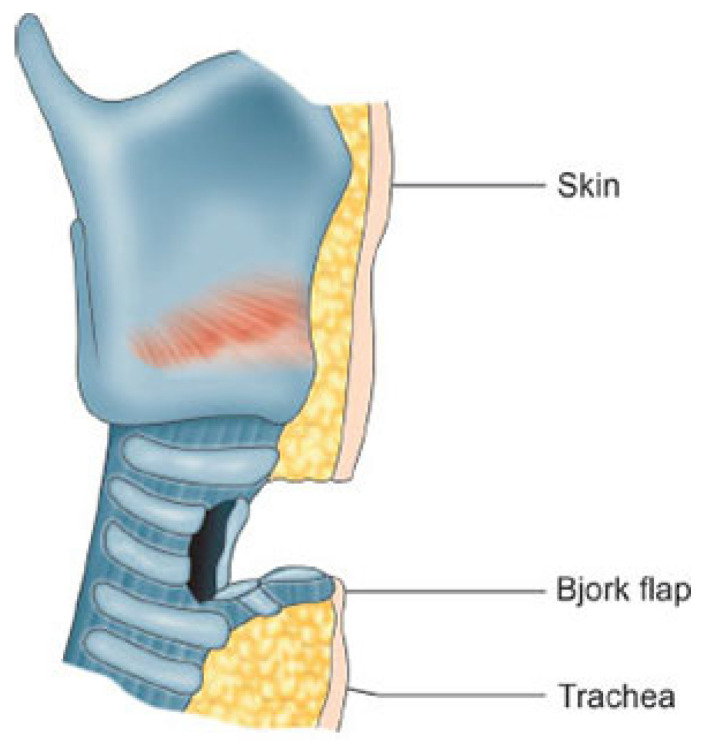
Bjork tracheostomy flap, lateral view.

Other techniques include creating a lower tracheal flap that interrupts only one tracheal ring, which is then sutured to the lower edge of the skin incision, while the remaining skin is sutured around the edge of the trachea (Bjork flap; Figure 9 and Figure 10).

Atlas of the oral and maxillofacial surgery clinics of North America 2010.

Textbook of Surgery for Dental Students by Sanjay Marwah

Less commonly used techniques include the creation of an upper tracheal flap (Eliachar technique), three-dimensional Z-plasty, and the “star plasty” (Koltai technique) [9].

**Figure 11:**
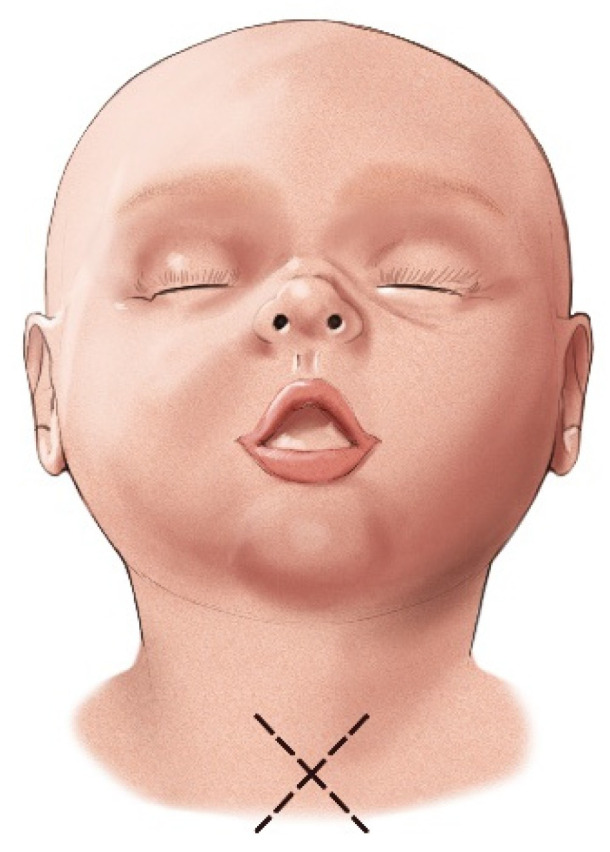
X-shaped skin incision.

**Figure 12:**
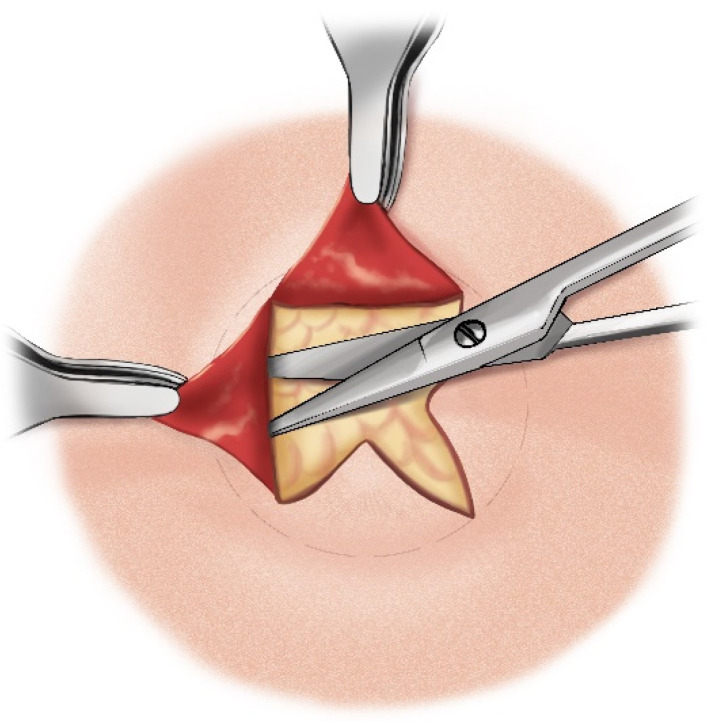
Triangular skin flap dissection.

**Figure 13:**
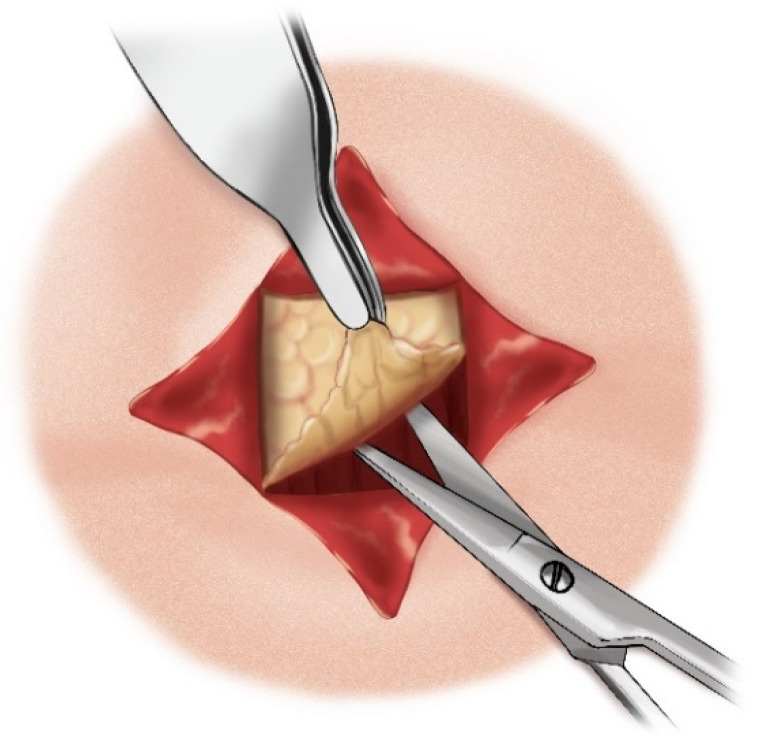
Adipose tissue dissection.

**Figure 14:**
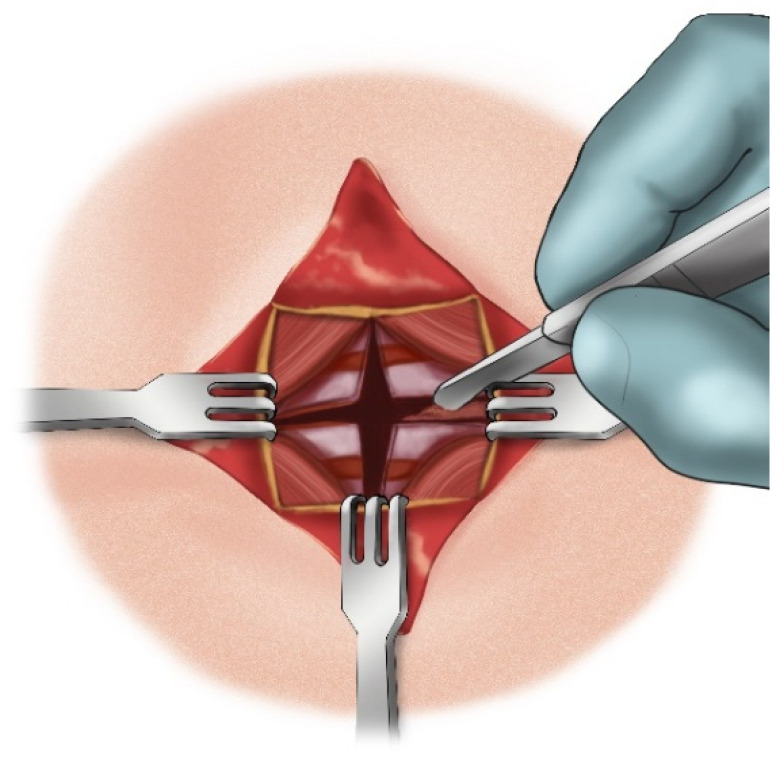
Incision of the trachea.

**Figure 15:**
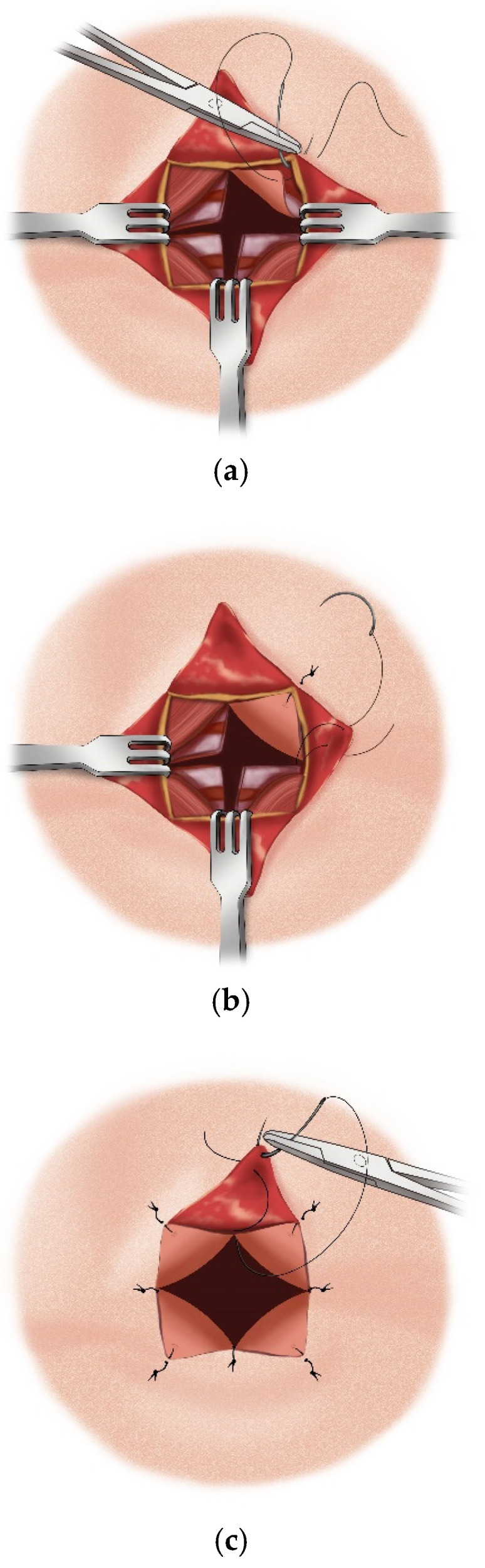
(**a**–**c**) Suturing of the tracheal and skin flaps.

The aforementioned “star plasty” technique by Koltai is not only a different type of tracheal plasty for creating a permanent tracheostomy opening but also an alternative variation of the entire classical tracheotomy process. The process is described in several steps as follows:

1. A cruciate or X-shaped neck skin incision is made midway between the cricoid cartilage and the jugular notch of the sternum (Figure 11). In children under one year of age, each skin incision forming the X should be 1 cm long. In older children, the incision length is proportionally increased, reaching up to 2 cm in adolescents.
2. The resulting triangular skin flaps are dissected from the underlying tissues using a dissecting scissors (Figure 12).
3. The subcutaneous adipose tissue covering the elongated neck muscles is removed (Figure 13).
4. The pretracheal connective tissues (pretracheal fascia) are dissected to expose the trachea.
5. Typically, the isthmus of the thyroid gland remains above the dissection field; however, in cases where it covers the trachea, it may be longitudinally divided, and the lobes of the gland sutured.
6. A vertical incision is made on the anterior tracheal wall, encompassing four tracheal rings, followed by a horizontal incision in the middle of the vertical incision (two tracheal rings above and two tracheal rings below), thus creating a shape resembling a plus sign (+) (Figure 14).
7. The triangular skin flaps and tracheal flaps are integrated at the periphery, using Vicryl 5.0 sutures. First, the tip of the right upper tracheal flap is sutured to the base of the right upper skin flap (Figure 15a), and then the base of the adjacent tracheal flap is sutured to the tip of the adjacent skin flap (Figure 15b). The remaining flaps are sutured sequentially in the same manner until the tracheostomy is formed. Since the skin and tracheal incisions diverge at an angle of 45°, the tips of the tracheal flaps fit into the indentations of the skin flaps, and the tips of the skin flaps tightly conform to the indentations of the tracheal flaps (Figure 15c).

A brief comparison of classical tracheotomy and Koltai’s tracheotomy is presented in Table 1.

Although tracheotomy is performed in the same manner in both children and adults, regardless of the surgical technique used, it is technically more challenging in childhood (especially under the age of one). This is due to the smaller and softer trachea and the closer proximity of the anatomical structures in the neck, which makes them more vulnerable to injury. A smaller surgical field also makes surgery more difficult.

## 6. Discussion

Previously, pediatric tracheotomy was primarily performed as a surgical procedure to overcome airway obstruction caused by severe infectious diseases [10,11]. Nowadays, it has increasingly become a planned surgical intervention for children with severe chronic illnesses [5], with a decreasing number of pediatric tracheotomy cases due to acute asphyxial incidents. An exception to this trend were cases of pediatric trauma that necessitated a tracheotomy.

A commonly performed surgical procedure in adults, every detail related to tracheotomy—from the indications and decision-making process to the surgical technique and postoperative care—has been perfected. In contrast, this procedure is performed significantly less often in children. This, in turn, leads to a number of shortcomings in its implementation, as well as to a certain number of unresolved issues primarily related to postoperative care, and to a much lesser extent, the surgical procedure itself. Publications on this topic are also relatively few and are based on the observation of a much smaller number of cases compared to adults. This international observation was also confirmed by our own experience; over the past 10 years, out of 24 pediatric tracheotomies performed in our center, 22 were planned due to the progression of a chronic disease, while only two were emergency procedures—one due to congenital cervical lymphangioma and one solely due to an acute inflammatory disease (COVID-19 infection). In contrast, during the same period, the average annual number of tracheotomies performed in adults is 46 (approximately 19.17 times more frequently).

For performing a tracheotomy in pediatric patients, the first step is usually obtaining informed consent from the parents. An exception is made in cases of progressing dyspnea, where the procedure is performed as an emergency intervention without waiting for parental consent based on vital indications.

The choice of the exact surgical technique for performing a tracheotomy is usually made in advance. However, in some cases, intraoperative modifications may occur, although they are generally minor. Most leading centers performing pediatric tracheotomies do not recommend the percutaneous approach, especially for children aged one year and younger. At our center, all pediatric tracheotomies have been performed using the classical open approach. A comparison of the characteristics of the two types of tracheotomy—classical and percutaneous—is presented in Table 2.

The intraoperative creation of a permanent or temporary tracheostomy is also subject to prior selection. Permanent tracheostomy is primarily performed in children with severe progressive and genetic diseases, where no significant improvement in the condition is expected. It is also preferred in children who are expected to be discharged from the hospital with a cannula and subsequently cared for at home, as it facilitates care and enhances security in maintaining free airways. Patients who are expected to require long-term mechanical ventilation and bronchoalveolar lavage are also indicated for permanent tracheostomy.

Each surgical technique is associated with a certain number of postoperative complications. A brief comparison of the complications related to the main types of tracheotomy (classical and percutaneous) and those associated with the most commonly used surgical techniques for forming a permanent tracheostomy is presented in Table 3.

Our modest experience with pediatric tracheotomy also led to the occurrence of postoperative complications—five cases of accidental decannulation in the early postoperative period, 10 cases of accidental decannulation in the late postoperative period, and three cases of peristomal granulation tissue that required surgical removal.

It should be noted that the choice of surgical technique also depends on the experience of the team performing the tracheotomy. At our center, the initial tracheotomies followed the classic steps for creating a temporary tracheostomy. Gradually, with accumulated experience and based on the challenges associated with managing this type of stoma, we transitioned to performing permanent tracheostomies in a significant number of patients, primarily in those with severe neurological diseases. The main surgical techniques used were the creation of an inferior flap (Björk tracheostomy flap) and suturing of the entire tracheal opening to the skin. Over the past 10 years, the statistics for our center have shown that 56% of tracheostomies performed in children were permanent.

It is extremely important to select and place the correct cannula size that is suitable for the patient during the procedure. In many cases, due to a lack of experience with pediatric tracheotomy, the largest possible diameter cannula is used. However, both our experience and global data suggest the opposite—optimal outcomes are achieved when the smallest cannula that ensures adequate pulmonary ventilation is used. Of course, like most medical principles, this one also has exceptions: a larger cannula is preferred to facilitate bronchoalveolar lavage and, in cases of mechanical ventilation, to minimize air leakage from the system.

The length of the cannula should extend at least 2 cm below the tracheostomy opening, and its tip should be positioned no closer than 1–2 cm above the carina of the trachea [6]. The appropriate cannula size for a child’s age can be determined using the following formula: for children older than one year, the inner diameter of the cannula (in millimeters) should be equal to the child’s age divided by 4, plus 4 mm. Another formula providing identical results is used in the table by Cole et al. to determine the most appropriate pediatric cannula size (Table 4). The obtained results can be compared with the sizing charts provided by different manufacturers of pediatric cannulas to select the best option.

In the most challenging cases, and when a cannula that meets the needs of a young patient cannot be found, an individual one can be manufactured. However, this can be done in the postoperative period, while during the surgical intervention, the most suitable available option is used.

**Figure 16:**
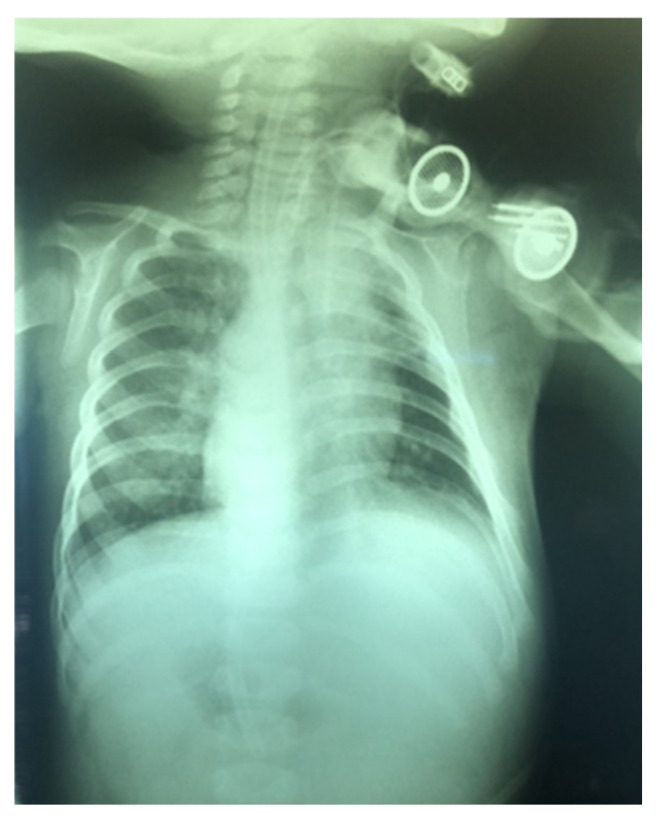
X-ray 4 h after cannula placement.

After a successful tracheotomy in childhood, several authors have recommended endoscopic examination of the trachea as a routine procedure [12]. In other centers, the examination is performed only if difficulties related to the tracheotomy, such as poor breathing through the cannula, bleeding, and difficulties in changing the cannula, arise [13]. In the absence of the necessary equipment or trained personnel, postoperative fibroendoscopy can be replaced by X-ray examination in the appropriate projection (Figure 16).

## 7. Conclusions

The described surgical approaches, established in international practice today, have achieved a high level of technical refinement. The choice of a specific approach depends on the child’s condition and the medical expectations regarding the progression of the disease that has led to the need for tracheotomy.

However, the existence of various operative approaches for performing tracheotomy in childhood is evidence of the lack of an ideal technique for its execution. The main requirements for such a technique would include technical simplicity, absence of tracheal secretions in the wound, easy postoperative cannula replacement, rapid stoma maturation with minimal maintenance, minimal tracheal deformation, and easy decannulation with minimal scar formation [11]. Each type of modern tracheotomy meets a certain number of these requirements, with the choice of the precise surgical technique depending on the individual needs of the young patients who require it.

Even when performed by an experienced team, tracheotomy in childhood can be accompanied by complications both during the procedure and in the postoperative period [14]. Proper preoperative preparation and postoperative care are essential for achieving a successful surgical outcome.

Given the small number of pediatric tracheotomies performed in individual centers, it would be beneficial to conduct a multicenter study and meta-analysis of the data in order to derive results from the observation of a larger number of patients.

## Figures and Tables

**Table 1:** Comparison ofclassical and Koltai’s tracheotomy.

Characteristic	Classical Tracheotomy	Koltai Tracheotomy
**Target Population**	Adults and children	Primarily, children (especially infants and young children)
**Purpose**	To secure the airway	To secure the airway with minimal trauma and improved stability
**Surgical Approach**	Open incision with horizontal cutting of tracheal rings	Vertical incision through 2–3 tracheal rings without excising cartilage
**Cannula Fixation**	Usually with neck ties	Cannula is stabilized by the shape of the incision—no sutures needed
**Complications (General)**	Higher risk of granulation, stenosis, and displacement	Lower risk of stenosis and improved cannula stability
**Postoperative Care**	More challenging for small children	Easier—reduced risk of accidental decannulation
**Healing after Decannulation**	Slower, often leaves a persistent fistula	Faster closure, often without the need for surgical revision

**Table 2:** Comparison of classical and percutaneous tracheotomy.

Characteristic	Surgical Tracheotomy (Classical)	Percutaneous Tracheotomy
**Technique**	Open surgical procedure with skin and tissue incision	Minimally invasive technique with puncture and dilation
**Setting**	Usually performed in the operating room	Typically performed in the intensive care unit
**Procedure Duration**	Longer (30–60 min)	Shorter (10–20 min)
**Anesthesia**	General or local	Usually local anesthesia with sedation
**Risk of Complications**	Higher—bleeding, infection, damage to adjacent structures	Lower—when performed correctly and on selected patients
**Suitable for**	Patients with anatomical anomalies, neck infections, tumors	Stable patients without contraindications
**Scar Visibility**	More noticeable	Less noticeable

**Table 3:** Comparison of complications of the most commonly performed types of tracheotomy.

Complication	Classical Tracheotomy	Björg Flap Tracheotomy	Koltai Tracheotomy	Percutaneous Tracheotomy
**Tracheal ** **Stenosis**	Common—especially with horizontal incision of rings	Rare—flap provides better anatomical stability	Rare—vertical incision minimizes trauma to the trachea	Rare—minimally invasive technique with minimal trauma to the trachea
**Granulation Around the Cannula**	Common in prolonged cannulation	Less frequent—flap stability reduces the chance of granulation	Less frequent—less traumatic technique and stable cannula	Less frequent—smaller wound, fewer chances for infection or granulation
**Cannula ** **Displacement**	More common—especially in children or restless patients	Rare—flap reduces the risk of displacement	Very rare—cannula is securely held by the incision shape	More common—can occur with improper placement or care
**Intraoperative Bleeding**	Possible—especially if thyroid or vessels are cut	Minimal—flap minimizes bleeding by avoiding major vessels	Minimal—minimally invasive technique avoids blood vessels	Very rare—minimally invasive approach with low bleeding risk
**Wound Site ** **Infection**	Possible with improper care	Less common—minimal incision and flap stability	Less common—smaller incision and better healing of the wound	Similar risk, but smaller wound and less external exposure
**Persistent ** **Tracheocutaneous Fistula**	Common after decannulation with prolonged cannulation duration	Less frequent—flap stability aids faster healing	Less frequent—faster closure after decannulation	Less frequent—minimal incision and faster healing

**Table 4:** Approximate size of the cannula for infants and children (Cole et al.).

Patient Age	Inner Diameter (mm)
Premature (less than 1000 g)	2.5
1000–2500 g	3.0
Newborn to 6 months	3.0–3.5
6 months to 1 year	3.5–4.0
1–2 years	4.0–5.0
Over 2 years	(Age in years +16)/4

## Data Availability

Data are available from the international sources mentioned in the references.
